# A novel Cre-enabled tetracycline-inducible transgenic system for tissue-specific cytokine expression in the zebrafish: CETI-PIC3

**DOI:** 10.1242/dmm.042556

**Published:** 2020-06-26

**Authors:** Sara Ibrahim, Arianna Harris-Kawano, Isra Haider, Raghavendra G. Mirmira, Emily K. Sims, Ryan M. Anderson

**Affiliations:** 1 Departments of Biochemistry and Molecular Biology, Indiana University School of Medicine, Indianapolis, IN 46202, USA; 2 Center for Diabetes and Metabolic Diseases, Indiana University School of Medicine, Indianapolis, IN 46202, USA; 3 Pediatrics, Indiana University School of Medicine, Indianapolis, IN 46202, USA; 4 Cellular and Integrative Physiology, Indiana University School of Medicine, Indianapolis, IN 46202, USA; 5 Medicine, Indiana University School of Medicine, Indianapolis, IN 46202, USA; 6 Kovler Diabetes Center and Department of Medicine, The University of Chicago, Chicago, IL 60637, USA

**Keywords:** Inflammation, Zebrafish, Beta cell, Cytokines, Pancreas

## Abstract

Maladaptive signaling by pro-inflammatory cytokines (PICs), such as TNFα, IL1β and IFNɣ, can activate downstream signaling cascades that are implicated in the development and progression of multiple inflammatory diseases. Despite playing critical roles in pathogenesis, the availability of *in vivo* models in which to model tissue-specific induction of PICs is limited. To bridge this gap, we have developed a novel multi-gene expression system dubbed Cre-enabled and tetracycline-inducible transgenic system for conditional, tissue-specific expression of pro-inflammatory cytokines (CETI-PIC3). This binary transgenic system permits the stoichiometric co-expression of proteins Tumor necrosis factor a (Tnfa), Interleukin-1 beta (Il1b) and Interferon gamma (Ifng1), and H2B-GFP fluorescent reporter in a dose-dependent manner. Furthermore, cytokine misexpression is enabled only in tissue domains that can be defined by Cre recombinase expression. We have validated this system in zebrafish using an *insulin*:*cre* line. In doubly transgenic fish, quantitative real-time polymerase chain reaction demonstrated increased expression levels of *tnfa*, *il1b* and *ifng1* mRNA. Moreover, specific expression in pancreatic β cells was demonstrated by both Tnfa immunofluorescence and GFP fluorescence. Cytokine-overexpressing islets elicited specific responses: β cells exhibited increased expression of genes associated with reactive oxidative species-mediated stress and endoplasmic reticulum stress, surveilling and infiltrating macrophages were increased, and β cell death was promoted. This powerful and versatile model system can be used for modeling, analysis and therapy development of diseases with an underlying inflammatory etiology.

This article has an associated First Person interview with the first author of the paper.

## Abstract (precis)

**Summary:** Here, we report a novel transgenic zebrafish system that permits tissue-specific, inducible and tunable expression of pro-inflammatory cytokines for modeling inflammatory diseases.

## INTRODUCTION

Pro-inflammatory cytokines (PICs) are signaling molecules that are primarily produced by immune cells (including activated macrophages and T cells) that coordinate and amplify certain immune responses. Dysregulated PIC activity is also involved in several disease processes, such as cell injury, infection, invasion and inflammation (Zhang and An, 2007). As such, pro-inflammatory cytokines play a critical role in diseases with an underlying maladaptive inflammatory pathology (Dinarello, 2000). For example, cytokines such as interferon gamma (IFNɣ), interleukin 1 beta (IL1β) and tumor necrosis factor alpha (TNFα; also known as TNF) can drive beta (β) cell dysfunction, damage and death in diabetes mellitus (Hatanaka et al., 2006; Dandona et al., 2004). This can potentially occur via pro-inflammatory cytokine-induced activation of intrinsic cellular signaling pathways, such as the generation of reactive oxygen species (ROS), leading to oxidative stress, or the accumulation of misfolded proteins, leading to endoplasmic reticulum (ER) stress (Wellen and Hotamisligil, 2005; Donath and Shoelson, 2011; Eriksson, 2007; Ambade and Mandrekar, 2012; Morito and Nagata, 2012). Furthermore, feedback amplification loops of cytokine signaling have been shown to further attract more innate immune cells and augment the innate immune response (Németh and Mócsai, 2016; Canna and Behrens, 2012). These pathways are important mediators of disease in islet injury observed in diabetes and also in tissue injury caused by inflammation, rendering pro-inflammatory cytokines crucial mediators and aggravators of many disease states.

Because pro-inflammatory cytokines play an essential role in progression of diabetes, cytokines are often used by supplementing cell culture media with a cytokine cocktail consisting of one or more cytokines to mimic a natural inflammatory environment *in vitro* (Weaver et al., 2012; Zhang et al., 2017). *In vivo* studies of cytokine expression either inject cytokines into the tail vein or intraperitoneally to induce a whole-body overexpression (Biesmans et al., 2015), or inject cytokines into tissues of interest such as the pancreatic duct (Valdez et al., 2016). However, approaches utilizing cytokine cocktails *in vivo* have several limitations, including inability for tissue-specific cytokine expression and inability to precisely titrate the level of induction of the cytokines. These limitations have been partially addressed by transgenic animal models. For instance, a transgenic mouse model of endotoxin-responsive TNFα expression *in vivo* exists (Keffer et al., 1991), although it does not offer tissue specificity. Another transgenic mouse misexpressing IFNɣ has been developed, but it does not allow for conditional regulated expression (Sarvetnick et al., 1990). Similarly, a zebrafish model of *in vivo* IL1β overexpression was recently established (Delgadillo-Silva et al., 2019). However, each of these models constitutively overexpresses the respective cytokine only in β cells and does not offer the versatility to induce expression in other tissues or the ability to titrate expression levels.

Since nearly 70% of human disease genes have a functionally similar homolog in the zebrafish (*Danio rerio*), it serves as a robust vertebrate model system in which to study conserved aspects of disease, while taking advantage of powerful attributes as an experimental system (Kulkarni et al., 2018). Furthermore, zebrafish are used to study diseases with an inflammatory component, such as diabetes, making them useful for studying pro-inflammatory cytokine signaling and their downstream effects (Kulkarni et al., 2018). Specifically, the conserved aspects of zebrafish innate immune responses relative to mammalian systems, together with the ease of genetic manipulation of the model system, make the zebrafish a powerful *in vivo* model for studying the effects of excessive cytokine induction (van der Vaart et al., 2012). To date, no models of tissue-specific, titratable pro-inflammatory cytokine induction in the zebrafish have been reported. In this study, we have developed a Cre-enabled and tetracycline-inducible transgenic system for conditional, tissue-specific expression of pro-inflammatory cytokines (CETI-PIC3), *tnfa*, *ifng1* and *il1b*. We demonstrate the utility of this versatile system to study the downstream effects of cytokine induction, which can be used to study multiple disease states and pathologies, using β cell-specific CETI-PIC3 fish as a proof of principle.

## RESULTS

### CETI-PIC3: a Cre-enabled tetracycline-inducible transgenic system for tissue-specific cytokine expression

**Fig. 1.**
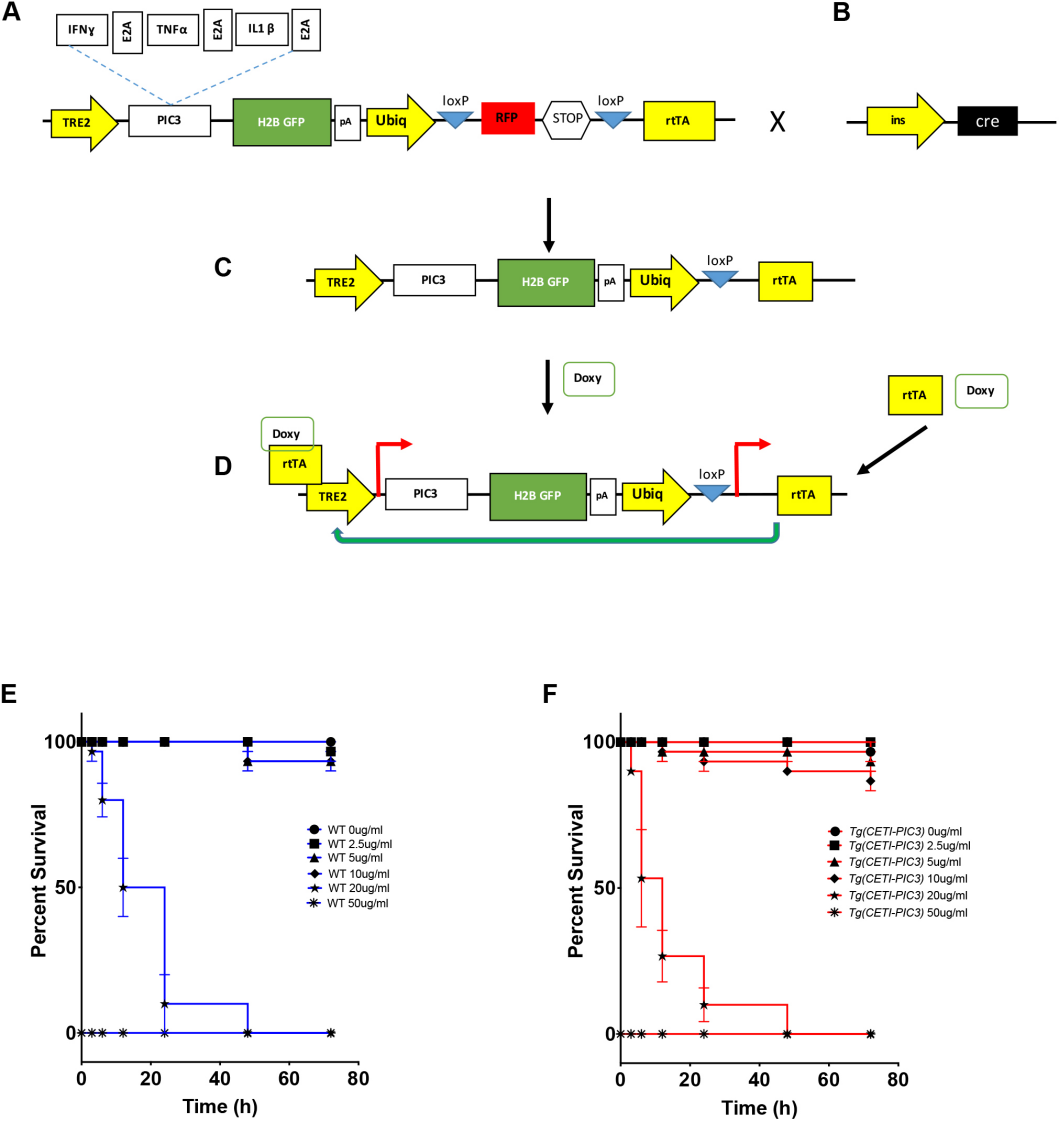
**Design of the CETI-PIC3 line.** (A) The genetic construct of the CETI-PIC3 line. A tetracycline-on (Tet-on) system is used to induce the three cytokines of interest. An H2B-GFP cassette is placed downstream of the cytokines as a marker to visually show that the cytokines have been induced. (B) This model takes advantage of Cre-lox systems to induce cytokines in a tissue-specific manner. (C) Any tissue-specific promoter driving a Cre cassette can be used to induce tissue-specific inflammation in this model after excision of the stop codon downstream of the RFP cassette. (D) When CET-PIC3 fish are crossed to a tissue-specific Cre and subsequently treated with doxycycline, there is dose-dependent induction of the cytokines in a tissue-specific manner. (E) Survival curve for wild-type (WT) embryos treated with different doses of doxycycline (0-50 µg/ml) for different time periods (0-72 h). (F) Survival curve for CETI-PIC3 embryos treated with different doses of doxycycline (0-50 µg/ml) for different time periods (0-72 h). There is no difference in survival for WT versus CETI-PIC3 doxycycline-treated embryos. *n*=10 embryos per dose/time. In all figures, data are mean±s.e.m.

**Fig. 2.**
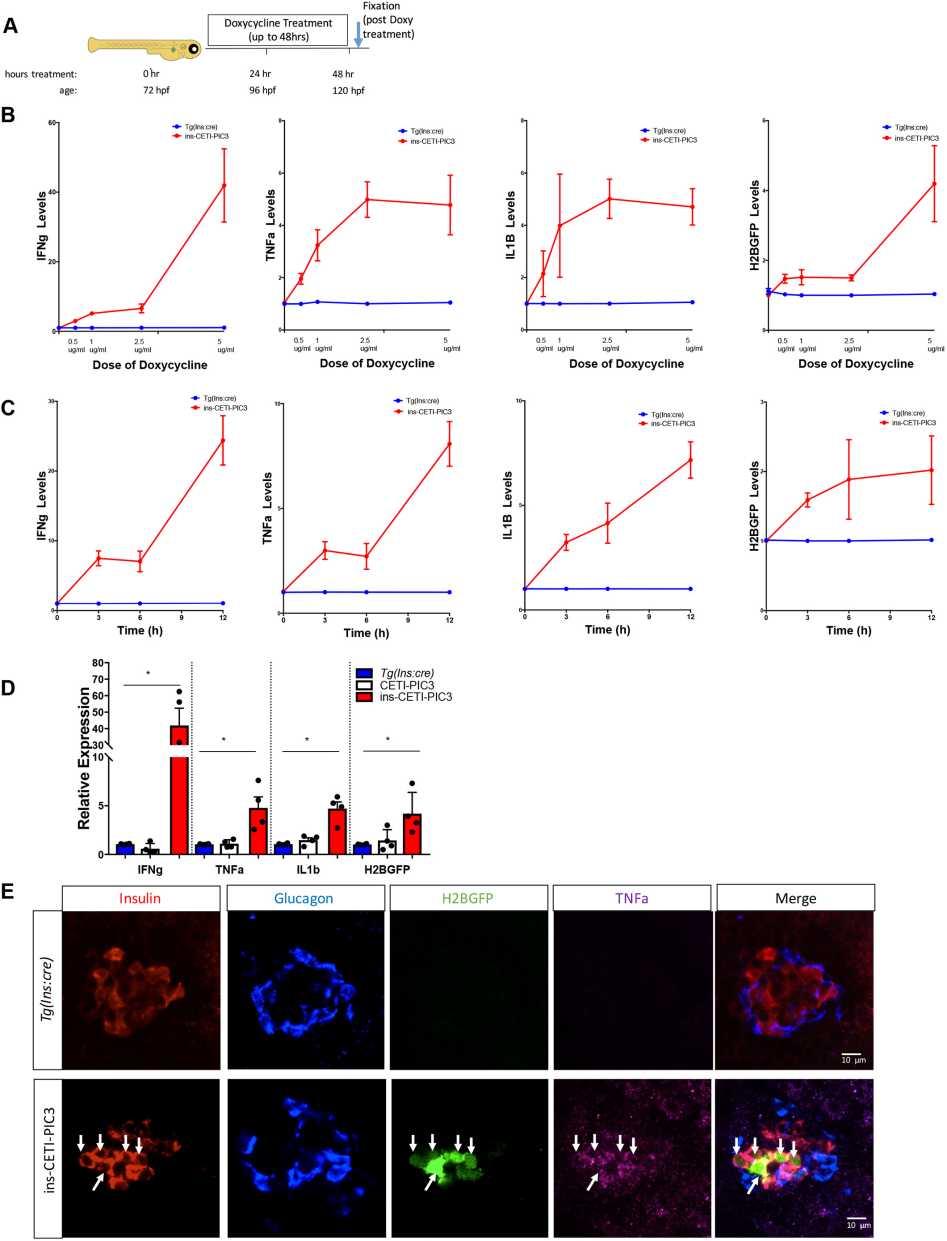
**The transcript levels of cytokines are increased and tissue-specific induction of cytokines is achieved within the induced ins-CETI-PIC3 model system.** (A) Schematic for the experimental procedure. All treatments were performed at 72 h post-fertilization (hpf). For B, C and D, RNA was subsequently isolated from the whole bodies of at least 15 embryos in each clutch and all analysis was performed on whole-body samples. (B) Dose response to 0, 0.5, 1 and 2.5 µg/ml doxycycline is shown for *tnfa*, *ifng1* and *il1b*, and *H2B-GFP*. (C) Time response to 5 µg/ml doxycycline is shown for up to 12 h after treatment with doxycycline. All experiments for D and E were performed in ins-CETI-PIC3 experimental embryos and clutch-mate control *Tg(ins:cre)* or CETI-PIC3 embryos treated with 5 µg/ml doxycycline for 48 h. (D) The levels of *tnfa*, *ifng1* and *il1b*, and *H2B-GFP* are all increased after doxycycline induction in the ins-CETI-PIC3 embryos compared to *Tg(ins:cre)* and CETI-PIC3 control embryos. *n*=4. *P<0.05 (one-way ANOVA). (E) After crossing the CETI-PIC3 line to the *Tg(ins:cre)* line, β cell-specific induction of the cytokines occurred, as seen by the H2B-GFP signal and Tnfa staining only in the Insulin-positive cells (arrows).

To study aspects of the pathogenesis of pro-inflammatory diseases, we have devised a novel genetic system to induce the expression of pro-inflammatory cytokines in a dose-dependent and tissue-restricted manner (Fig. 1). IFNɣ, TNFα and IL1β are three pro-inflammatory cytokines that are mediators of cellular damage in multiple diseases. The encoding sequences of the zebrafish orthologs of each of these cytokines and a nuclear green fluorescent protein H2B-GFP were linked together with viral 2A elements to form the pro-inflammatory cytokines 3 (PIC3) cassette that was then cloned downstream of a tetracycline-dependent promoter (TRE2) to create the CETI-PIC3 line (Fig. 1A). In this multi-gene expression system, the three cytokines and a fluorescent cellular indicator are designed to be expressed at similar levels (de Felipe and Ryan, 2004; Donnelly et al., 2001). In addition, a mechanism for restricting expression to particular cell types was incorporated into our expression system via Cre-loxP elements (Fig. 1B). Specifically, a ubiquitin promotor (Mosimann et al., 2011) drives the expression of a red fluorescent protein (RFP, mCherry) that is flanked by loxP sequences, generating a ubiquitous red ‘baseline’ fluorescence throughout the transgenic animal. When CETI-PIC3 fish are crossed to a tissue-specific Cre, the recombinase activity excises the RFP and STOP sequences, permitting expression of reverse tetracycline trans activator (rtTA) and thereby enabling the inducibility component in a tissue-specific pattern (Fig. 1C). Upon subsequent exposure of the transgenic zebrafish to doxycycline, the drug binds and activates rtTA, which consequently drives expression at the tetracycline-dependent promoter (Meerbrey et al., 2011). This combination of elements from both the tetracycline-on (Tet-on) and Cre-lox systems with viral 2A sequences permits multigene expression of these three pro-inflammatory cytokines in an inducible and tissue-specific manner (Fig. 1D). To determine the maximal dosing of doxycycline for transgene induction without toxicity, transgenic CETI-PIC3 animals and control wild-type (WT) embryos were incubated with a range of doxycycline doses (0-50 µg/ml) for varying durations (0-72 h), and survival curves were plotted (Fig. 1E,F). Lethality was observed at higher doses, but there was no difference in survival between the CETI-PIC3 embryos and WT embryos across all treatment regimens tested; this indicates that the lethality is due to the toxicity of high doxycycline dosing, not leaky Cre-independent expression of rtTA and/or non-specific PIC3 cassette expression. Next, to confirm that the CETI-PIC3 system is competent to drive the induction of the PIC3 pro-inflammatory cytokine expression cassette in a titratable manner, the levels of *tnfa*, *ifng1* and *il1b*, and *H2B-GFP* mRNA were measured using quantitative real-time polymerase chain reaction (qRT-PCR) analysis of whole zebrafish larvae. These measurements reflect both transgene-induced cytokine expression and endogenous message. Zebrafish were either treated for 48 h with varying doses of doxycycline or with 5 µg/ml doxycycline for varying durations (Fig. 2A). For this, the *Tg(CETI-PIC3)^iu15^* fish were crossed to *Tg(ins:cre)^s924^* fish to generate doubly heterozygous (ins-CETI-PIC3) experimental embryos. As a control, single-heterozygous *Tg(ins:cre)* embryos and single-heterozygous CETI-PIC3 embryos were treated identically. In the double transgenics, we measured increased levels of *tnfa*, *ifng1* and *il1b*, and *H2B-GFP* that were correlated to doxycycline dose, which ranged from 0 to 5 µg/ml (Fig. 2B). In addition, we measured steadily increasing levels of these four coding sequences at time points progressing from 0 to 12 h in response to constant dose of 5 µg/ml doxycycline (Fig. 2C). Based on these results, we used a dose of 5 µg/ml doxycycline for the remainder of our experiments because it not only enabled a relatively high induction of cytokines, but also permitted embryo survival with 48 h of treatment. Under these dosing parameters, the levels of all cytokines and H2B-GFP were increased by 5- to 40-fold in the doxycycline-treated ins-CETI-PIC3 embryos compared to *Tg(ins:cre)* and CETI-PIC3 controls (Fig. 2D).

We next checked to ensure that cytokine expression was enabled only in specific tissues. The *Tg(CETI-PIC3)^iu21^* allele was again combined with the *Tg(ins:cre)* line. Here, we observed β cell-specific expression of the cytokine cassette in ins-CETI-PIC3 embryos treated with doxycycline (Fig. 2E). This was evidenced by nuclear H2B-GFP expression as well as by Tnfa immunostaining within the Insulin-positive cells (Fig. 2E, arrows). In addition, tissue-specific expression of CETI-PIC3 was observed using pancreatic delta cell-specific (*sst2*) and hepatocyte-specific (*fabp10*) promoters (Fig. S2).

**Fig. 3.**
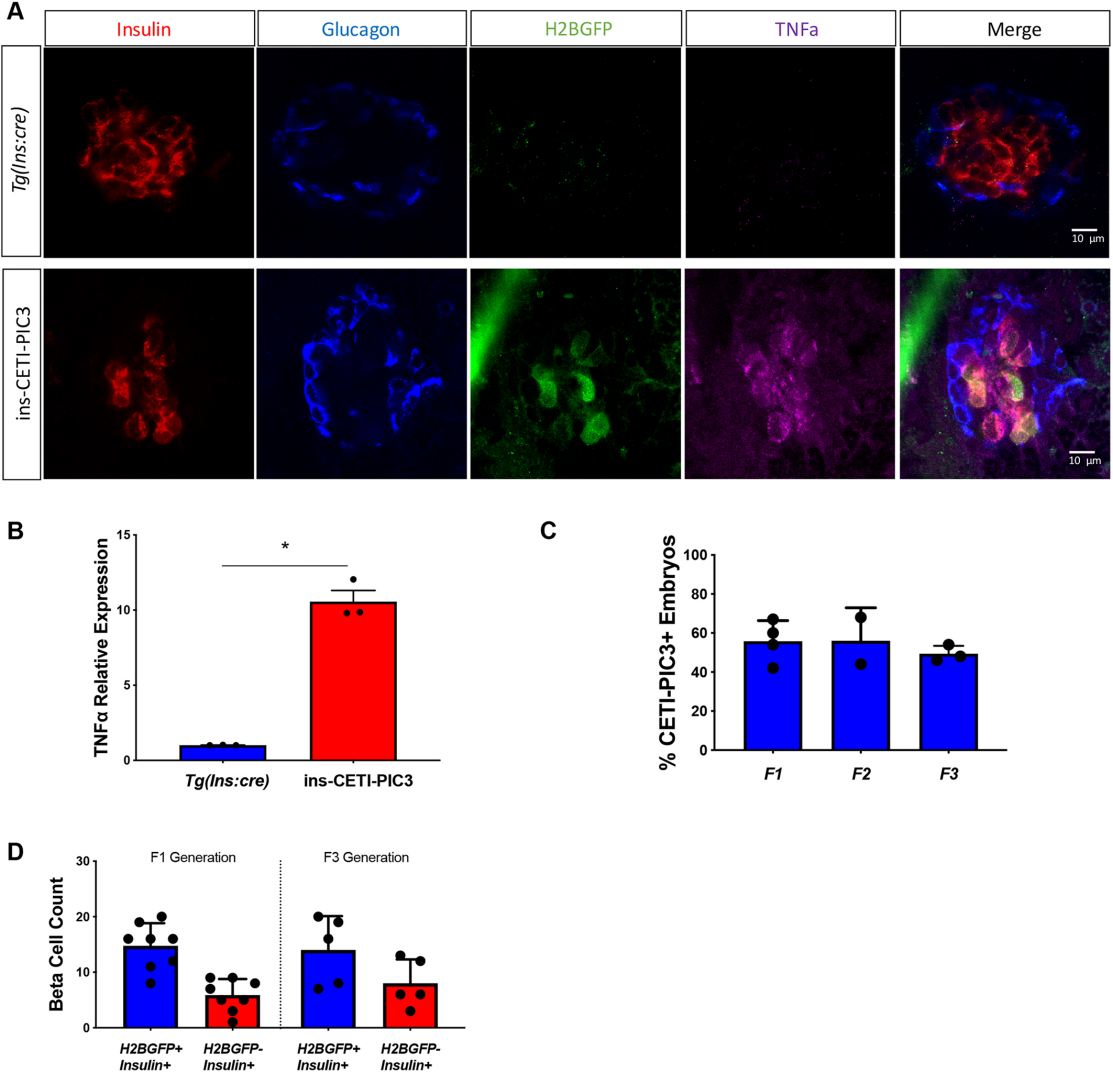
**The efficiency and effects of the CETI-PIC3 system** **are** **preserved in subsequent generations.** (A) Embryos from an F3 generation of the CETI-PIC3 line were crossed to the *Tg(ins:cre)* line. β cell-specific induction of H2B-GFP was observed and Tnfa staining was enriched within β cells. (B) qRT-PCR analysis demonstrates increase in *tnfa* transcript levels in this generation of embryos. *n*=3. *P<0.05 (parametric *t*-test). (C) The percentage of transgenic embryos from each generation, up to the F3 generation. (D) Quantification of Insulin^+^ H2B-GFP^+^ double-positive cells and Insulin^+^ H2B-GFP^−^ single-positive cells in ins-CETI-PIC3 embryos from F1 and F3 generations to show how responsive CETI-PIC3 embryos are to *insulin:cre*.

Lastly, there is the potential for diminished transgene expression in subsequent generations of newly founded transgenic lines generated with the Tol2 transposon system. This may be due to silencing mechanisms (Goll et al., 2009) or segregation of multiple insertions. To test the robustness of this new line, we examined F3 ins-CETI-PIC3 progeny for Tnfa and H2B-GFP expression after doxycycline treatment, and found that inducibility was maintained (Fig. 3A). Moreover, qRT-PCR demonstrated that the increase in *tnfa* transcript levels was also preserved in the F3 embryos (Fig. 3B). To test for multiple segregating alleles in the transgenic line, heterozygous CETI-PIC3 fish were crossed to WT fish and the percentage of transgenic embryos was quantified in each generation through F3 (Fig. 3C). No differences were observed between the generations. Lastly, we compared the efficiency of labeling β cells with H2B-GFP expression in F1 and F3 generations (Fig. 3D). Again, no differences were observed between the generations.

### Cytokine induction in ins-CETI-PIC3 embryos leads to increased islet macrophage surveillance and infiltration

**Fig. 4.**
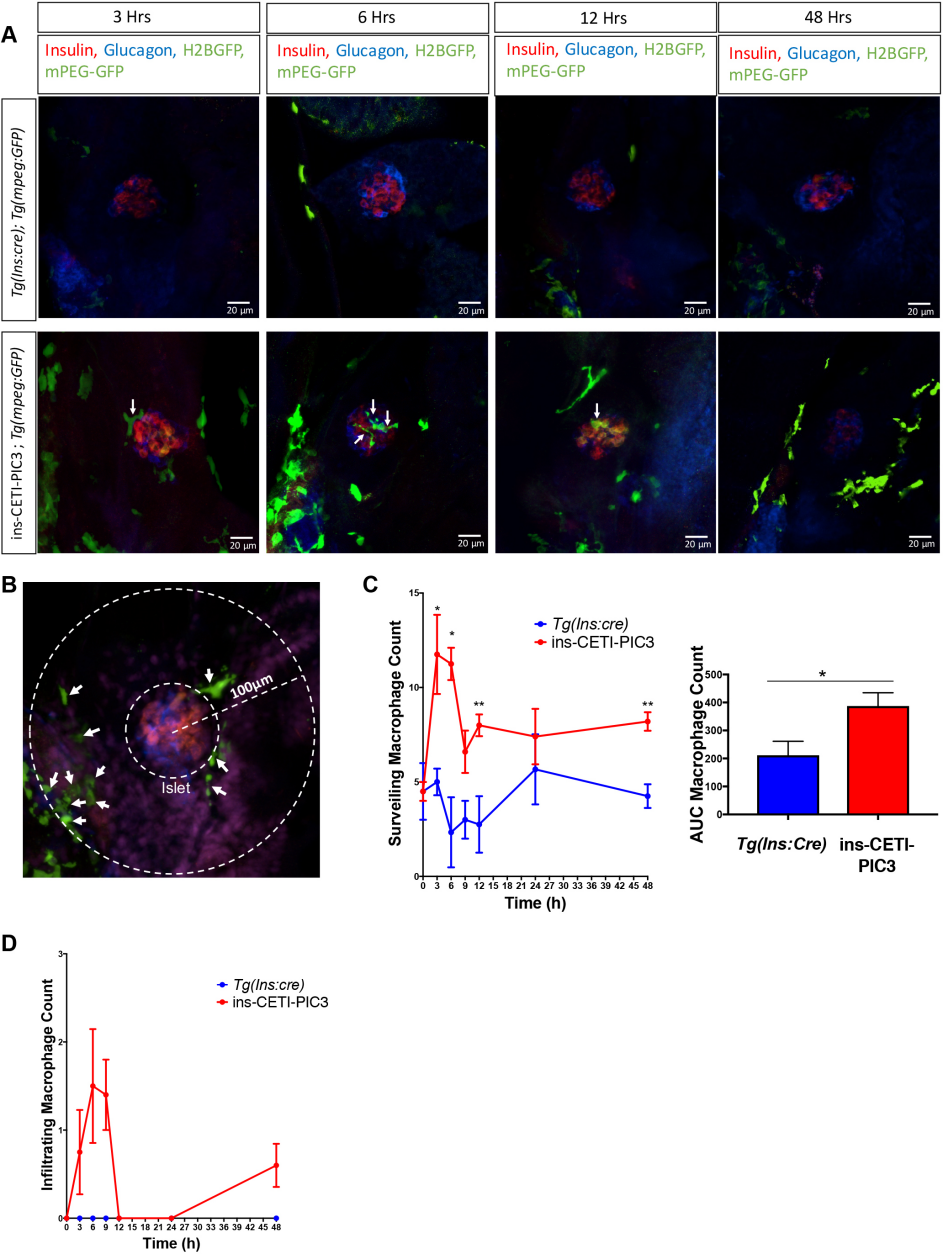
**Cytokine-induced embryos have increased macrophage surveillance and infiltration compared to clutch-mate controls.** (A) ins-CETI-PIC3 embryos were crossed to the *Tg(mpeg:GFP)* line that marks macrophages with a GFP tag. *CETI-PIC3;ins:cre*;*Tg(mpeg:GFP)* triple-positive embryos were compared to *Tg(ins:cre);Tg(mpeg:GFP)* control embryos. All embryos were treated with 5 µg/ml doxycycline for different time periods (0-48 h). Infiltrating macrophages are marked with arrows. (B) Schematic for how macrophages were counted. All macrophages within a 100-μm radius of the center of the islet were counted. All macrophages within that radius but outside the islet itself were considered surveilling macrophages (arrows) and all macrophages within the islet were considered infiltrating macrophages. (C) ins-CETI-PIC3 embryos display increased surrounding surveilling macrophages in a time-dependent manner. *n*=4-5 embryos. *P<0.05, **P<0.01 (parametric *t*-test). (D) Areas under the curves were calculated to quantify macrophage numbers for multiple embryos and showed that increased infiltrating macrophages were observed in ins-CETI-PIC3 embryos in a time-dependent manner. *n*=4-5 embryos.

In pathogenic contexts such as diabetes, the maladaptive expression of cytokines is associated with immune cell infiltration, cellular dysfunction and cell death. Cytokine-induced damage has been associated with the recruitment of immune cells such as macrophages to the site of insult (Arango Duque and Descoteaux, 2014). Macrophages are an innate immune cell type that secrete cytokines and are also very responsive to cytokine signaling (Parameswaran and Patial, 2010). To visualize the effects of cytokine induction on macrophage recruitment to the site of insult in ins-CETI-PIC3 embryos, we used the *Tg*(*mpeg:GFP)* transgenic line, in which macrophages are marked by green fluorescence (Ellett et al., 2011) (Fig. 4A,B). In the doxycycline-induced triply heterozygous embryos, we quantified surveilling macrophages that were within a 100-µm radius of the islet (Fig. 4C). We observed an increase in the number of these surveilling macrophages within the islet region beginning at the 3-h time point after the addition of doxycycline. Furthermore, infiltrating macrophages, usually absent from this region in control islets, appeared in the islet in a time-dependent manner after doxycycline treatment in ins-CETI-PIC3 embryos compared to treated *Tg*(*ins:cre*) clutch-mate controls (Fig. 4D).

### Cytokine induction in ins-CETI-PIC3 embryos leads to oxidative and ER stress and hyperglycemia

**Fig. 5.**
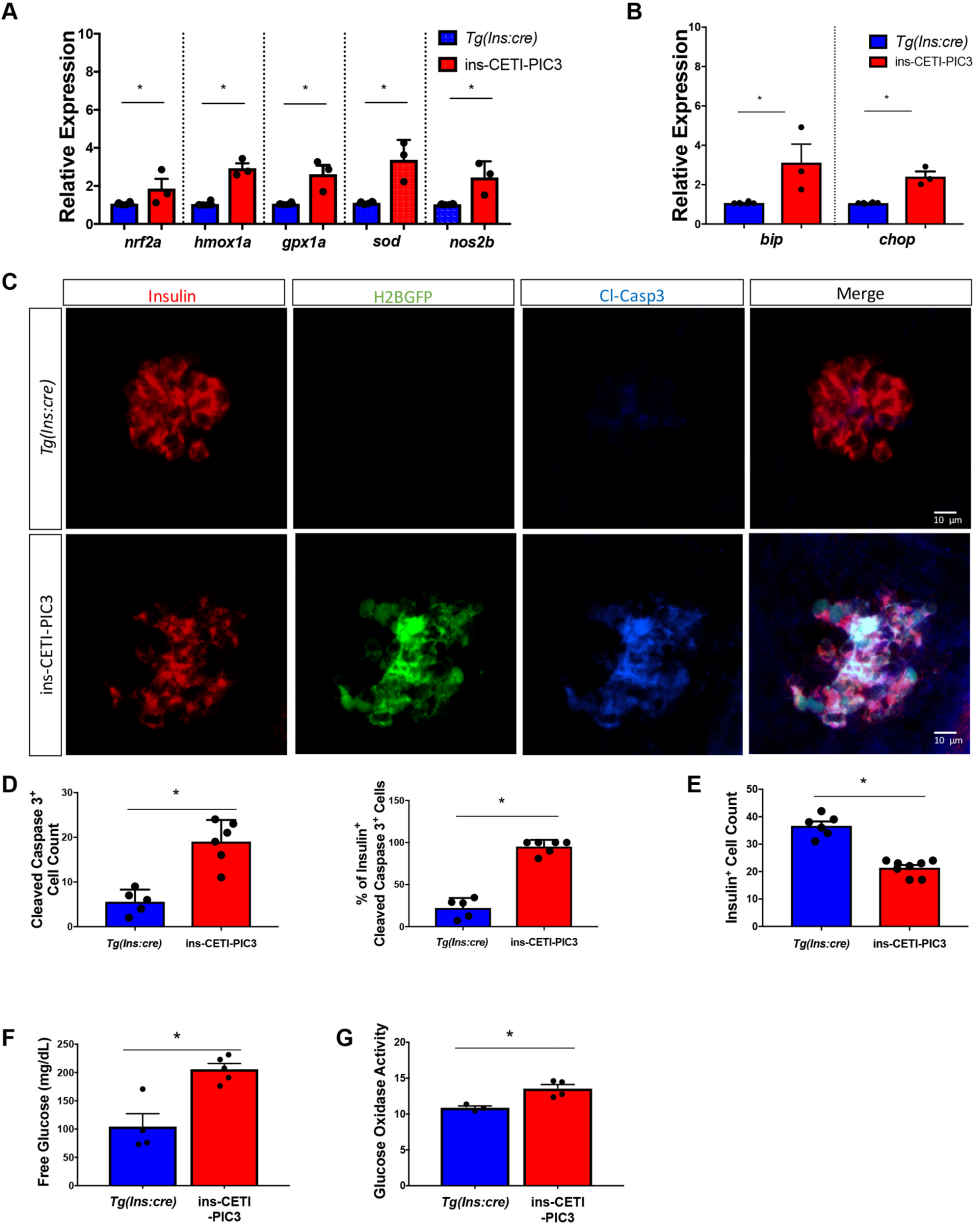
**Cytokine-induced embryos display increased transcript levels for markers of ROS-mediated and ER stress, and increased β cell death, and are hyperglycemic compared to clutch-mate controls.** All experiments in these panels were performed in ins-CETI-PIC3 experimental embryos and clutch-mate control *Tg(ins:cre)* embryos treated with 5 µg/ml doxycycline for 48 h. (A) Several members involved in the ROS-mediated stress pathway – such as *nrf2a*, *hmox1a*, *gpx1a*, *sod* and *nos2b* – were also increased after induction of the pro-inflammatory cytokines *n*=3. *P<0.05 (parametric *t*-test). (B) Members of the ER stress pathway, such as *bip* and *chop*, were increased following cytokine induction. *n*=3. *P<0.05 (parametric *t*-test). (C) There is increased Cleaved caspase 3 staining in ins-CETI-PIC3 embryos compared to *Tg(ins:cre)* controls. (D) The number of Cleaved caspase3^+^ cells per islet is increased in the ins-CETI-PIC3 embryos. *n*=6. *P<0.05 (parametric *t*-test). (E) The Insulin^+^ cell count is decreased in the ins-CETI-PIC3 embryos. (F) Free glucose levels in ins-CETI-PIC3 embryos are higher than those in *Tg(ins:cre)* controls. *n*=5. *P<0.05 (parametric *t*-test). (G) Glucose oxidase activity, an indicator of free blood glucose, is higher in ins-CETI-PIC3 embryos. *n*=4. *P<0.05 (parametric *t*-test).

Signaling through IL1β, TNFα and IFNɣ pathways can lead to downstream activation of cellular stress pathways. ROS are regulators of inflammatory signaling and can mediate downstream proteasome activity, antioxidant gene transcription, cytokine secretion and inflammasome activation (Forrester et al., 2018). Consistent with increased generation of ROS, the mRNA levels of *nuclear* *factor erythroid 2-related factor 2* (*nrf2a*; also known as *nfe2l2a*), *nitric oxide synthase 2* (*nos2b*), *heme oxygenase* (*hmox1a*), *glutathione peroxidase* (*gpx1a*) and *superoxide dismutase* (*sod*; also known as *sod1*) are each significantly increased in ins-CETI-PIC3 embryos induced with doxycycline (Fig. 5A). Furthermore, in many pathological settings, inflammation can result in sustained activation of the unfolded protein response (UPR) and trigger ER stress, exacerbating impacts of the inflammatory cascade (Zhang and Kaufman, 2008). Consistent with activation of ER stress signaling, we measured increased expression of the ER stress markers *binding immunoglobulin protein* (*bip*; also known as *hspa5*) and *C/EBP homologous protein 3* (*chop*; also known as *ddit3*) in induced ins-CETI-PIC3 embryos after exposure to doxycycline (Fig. 5B).

### Cytokine induction leads to β cell dysfunction and death in ins-CETI-PIC3 embryos

Sustained or high-dose inflammatory cytokine induction in β cells can result in apoptosis (Collier et al., 2011). Indeed, the observed increase in islet macrophage recruitment is consistent with increased β cell death due to cytokine expression. To determine whether β cell death was induced, we used immunostaining for Cleaved caspase 3 (Casp3*) to directly visualize apoptotic cells (Gown and Willingham, 2002). We found that ins-CETI-PIC3 embryos exhibited a substantial increase in the quantity of Casp3*-positive cells within the islet (Fig. 5C,D). Importantly, Casp3* staining was confined to the Insulin-positive β cells. Consistent with β cell loss, we also observed a decrease in total Insulin^+^ cell counts in the ins-CETI-PIC3 embryos (Fig. 5E).

**Fig. 6.**
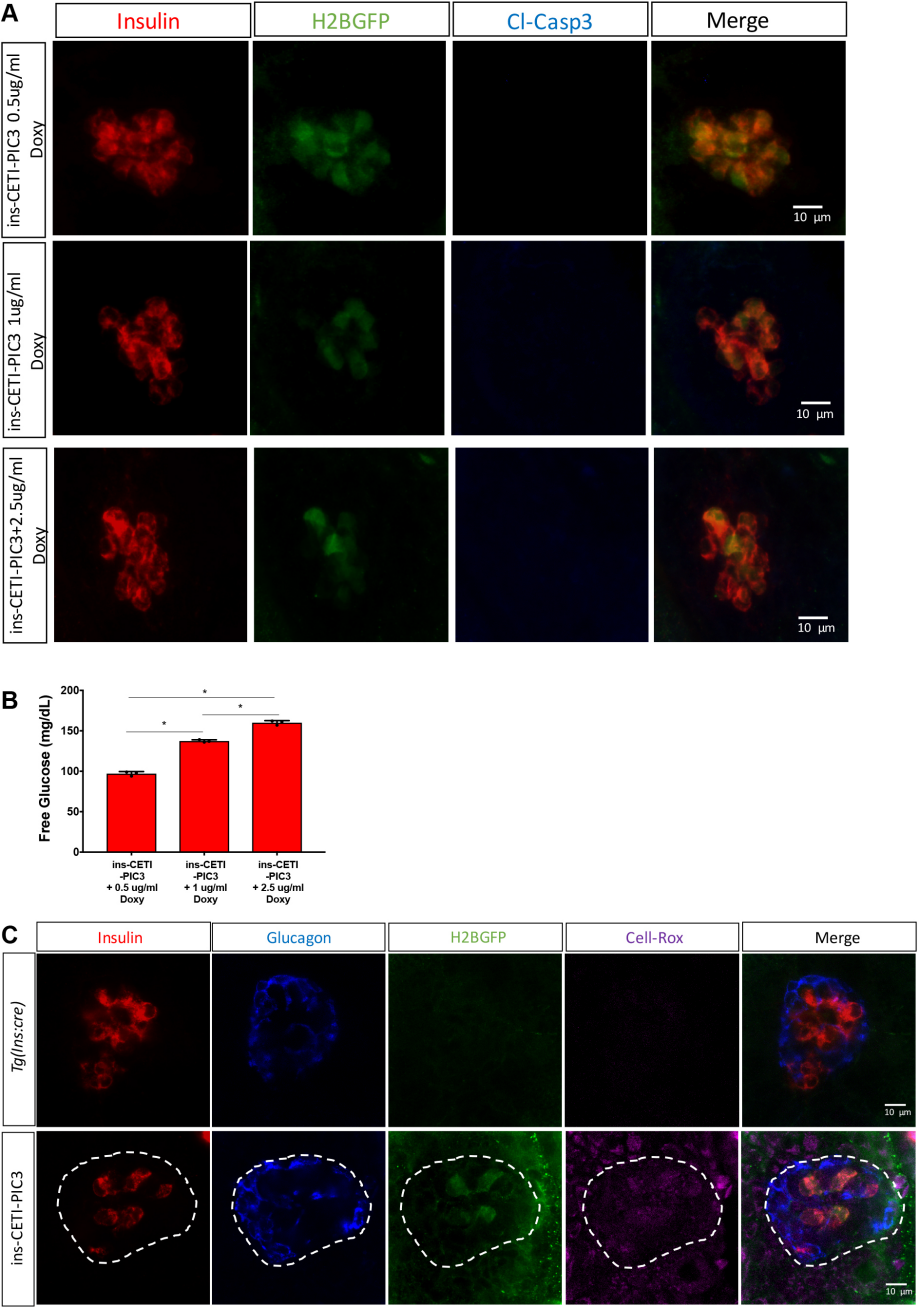
**Hyperglycemia is induced without overt β cell death and ROS-mediated stress is seen in surrounding cells of the islet.** (A) After treatment with 0.5, 1 and 2.5 µg/ml doxycycline, there was no subsequent increase in Casp3* expression in these embryos. (B) A hyperglycemic phenotype was observed in embryos treated with 1 µg/ml and 2.5 µg/ml doxycycline. *n*=3. *P<0.05 (individual *t*-tests). (C) The expression of CellRox Deep Red is enriched in β cells and is also observed in surrounding cells of the islet (outlined by dashed lines).

Previous studies have demonstrated that the induction of pro-inflammatory cytokines in β cells can cause systemic hyperglycemia as well as β cell death (Berchtold et al., 2016; Montane et al., 2014). We measured free glucose levels in ins-CETI-PIC3 embryos and *Tg(ins:cre)* controls that had been treated with 5 µg/ml doxycycline for 48 h and found that induction of pro-inflammatory cytokines in the β cells indeed resulted in hyperglycemia (Fig. 5F). To independently confirm hyperglycemia in these ins-CETI-PIC3 animals, we also measured glucose oxidase enzymatic activity. Consistent with our free glucose measurements, glucose oxidase activity was elevated in ins-CETI-PIC3 animals relative to *Tg*(*ins:cre*) clutch-mate controls (Fig. 5G). Hyperglycemia can result from either β cell dysfunction or death (Weir and Bonner-Weir, 2004). To determine whether cytokine induction in our model could produce a hyperglycemic/dysfunctional phenotype without overt β cell death, we titrated the dose of doxycycline and stained for Casp3*. In Fig. 6A and B, we show that animals treated with 0.5, 1 and 2.5 µg/ml doxycycline showed hyperglycemia without an associated increase in Casp3* expression in these embryos.

Although this ins-CETI-PIC3 gene expression system drives cytokine induction in a tissue-specific manner, as evidenced above, cytokine expression and activity may not be limited solely to β cells after induction for 48 h. As seen in Figs 2E and 3A, Tnfa expression was increased within β cells but was not limited to them. To assess whether a non-autonomous ‘bystander effect’ is also induced in adjacent cells, we stained doxycycline-induced animals with CellRox Deep Red, a marker for oxidative stress, which is elevated with inflammatory cytokine signaling (Kulkarni et al., 2018). We found that expression of CellRox Deep Red was enriched not only in β cells, but also in cells surrounding the islet (Fig. 6C).

## DISCUSSION

### Mechanism of cell damage by the CETI-PIC3 line

Pro-inflammatory cytokines have been shown to play a crucial role in the pathophysiology of several diseases and in numerous malignant processes. Although studying the role of pro-inflammatory cytokines is critical in understanding any disease with an underlying inflammatory etiology, the current model systems available to study pro-inflammatory cytokines *in vivo* are lacking. The most recent model generated to study pro-inflammatory cytokines *in vivo* in zebrafish does not allow for inducible control of cytokine expression, only induces a single cytokine (IL1β) and only works in a pancreatic β cell-specific manner (Delgadillo-Silva et al., 2019). Here, we have developed a versatile tissue-specific and dose-dependent model of cytokine induction in an *in vivo* zebrafish system. Phenotyping after induction of cytokine signaling showed increased activation of downstream signaling pathways, such as ROS signaling pathways and ER-mediated stress pathways. Furthermore, our model exhibited macrophage infiltration into regions of cytokine-misexpressing cells, consistent with prior work showing that cytokine production by other inflammatory conditions – such as diabetes, bacterial infection and cancer – induce innate immune responses such as activation of macrophages and neutrophils (Mizgerd et al., 2001; Martinez et al., 2008).

A reinforcing feedback activation loop of cytokine signaling and expression has previously been observed, whereby the levels of pro-inflammatory cytokines are increased, feeding a signaling cascade that subsequently augments the expression levels and activities of the triggering cytokines. For example, cytokines signal to recruit immune cells, including macrophages and T cells, which activates these cells, causing them to produce more cytokines (Canna and Behrens, 2012; Donath et al., 2013). The macrophages that are recruited to the site of insult will phagocytose diseased and damaged cells, leading to their programmed cell death (Hirayama et al., 2017). One of the critical cytokines produced by macrophages is IFNɣ (Darwich et al., 2009). This may explain the high expression levels of *ifng1* RNA, which were approximately eight times greater than the expression levels of the other cytokines and H2B-GFP comprising the PIC3 cassette.

The CETI-PIC3 system increases the levels of three pro-inflammatory cytokines (IFNɣ, TNFα and IL1β). These three pro-inflammatory cytokines in combination are elevated in several disease processes such as diabetes, pulmonary tuberculosis, and allograft and xenograft rejection (Cavalcanti et al., 2012; Wachlin et al., 2003; Batten et al., 1996). However, there are disease processes that do not exhibit an increase in all three cytokines in a stoichiometric manner (Zhang and An, 2007; Dinarello, 2000; Strober and Fuss, 2011; Monastero and Pentyala, 2017). Nevertheless, it is understood that there are complex signaling pathways and feedback mechanisms between cytokines in which one cytokine can lead to the expression of another cytokine (Leonard and Lin, 2000). Furthermore, multiple cytokines across several disease models exert their actions by signaling though common pathways such as the JAK-STAT and the NFκB signaling pathways (Zha et al., 2017). Here, we sought to develop a system in which we increase levels of three well-known cytokines in inflammatory pathology to develop a tool that will enable a better method to study inflammation and inflammatory diseases.

### Intricacies of the CETI-PIC3 system

Our model utilizes a two-part Tet-on system that is composed of a normally inactive Tet transcriptional activator that is activated in the presence of doxycycline, and a minimal promoter that is bound by the activated Tet to induce gene expression. This binary system has two components that are critical for inducing genes of interest: an inducible promoter upstream of the genes of interest (Zhang and An, 2007) and a constitutively expressed trans-activator that can be bound by doxycycline to mediate the activation of the inducible promoter (Dinarello, 2000). The expression level of the induced genes of interest depends on the strength of the promoter, ubiquitin in this case, that drives the transcriptional activator (Zabala et al., 2004). As there are multiple components in this expression system that must be sequentially transcribed, translated, folded and processed, it is possible that within a given cell there is a slight delay in the expression of the PIC3-GFP cassette compared to the expression of the Cre or other endogenous markers of that cell type. For instance, the transcription of endogenous *insulin* and *Cre* might be expected to initiate simultaneously because they depend on versions of the same promoter. However, before H2B-GFP reporter fluorescence is observable, the stop sequence of the PIC transgene must be excised by Cre, and the Tet activator protein must be transcribed, translated, activated and then translocated to the Tet-response element. This will activate transcription of PIC3-H2B-GFP, which finally will be translated and processed. A relative delay, while not necessarily dependent on distance between the components of the transgene, would need to be empirically determined. However, the presence of Insulin^+^ cells that are not H2B-GFP^+^ after induction is consistent with such a delay.

### Utility of CETI-PIC3 zebrafish system as a disease model

The use of this CETI-PIC3 model system to induce expression of pro-inflammatory cytokines in cells of the pancreatic islet to study both type 1 and type 2 diabetes is apparent. However, pro-inflammatory cytokines are important in the pathophysiology of many diseases, including atherosclerosis, inflammatory bowel disease, cardiovascular disease, nephritis, sepsis, cancer and arthritis. This CETI-PIC3 system can be used to study aspects of inflammatory disease for which an appropriate tissue-specific Cre is available. Alternatively, some diseases, such as sepsis, have systemic inflammation and this model can be crossed to a ubiquitous Cre to study whole-body inflammation. Some diseases, such as obesity, have also been shown to have systemic low levels of cytokine elevations. To model such diseases, low concentrations of doxycycline could be used to generate chronic cytokine induction.

Zebrafish have several advantages over rodents as model organisms for studying cytokine induction. The optical transparency of zebrafish along with its short life cycle allows for observation of the zebrafish non-invasively over time. This model is very flexible due to the added possibility of precisely modulating cytokine induction using doxycycline dosing and timing. This flexibility will facilitate future drug discovery and testing in conjunction with other zebrafish models that have previously been used for drug screening (Andersson et al., 2012). In conclusion, our work validates a novel zebrafish system for cell-specific pro-inflammatory cytokine activation. This system has widespread applicability for future work requiring tissue-specific study of pro-inflammatory diseases.

## MATERIALS AND METHODS

### Zebrafish husbandry and strains

All animal work was approved by the Indiana University School of Medicine Institutional Animal Care and Use Committee (IACUC) and carried out in accordance with IACUC standards. Zebrafish (*Danio rerio*) embryos were spawned and raised under standard laboratory conditions at 28.5°C. To generate the CETI-PIC3 line, a Gibson assembly approach was followed using multiple vector components. A PIC3 expression cassette comprised of zebrafish mRNA sequences for the cytokines *ifng1* (NCBI: NM_212864.1), *tfna* (NCBI: NM_212859.2) and *il1b* (NCBI: NM_212844.2), which were connected with intervening E2A sequences, was ordered as a single cDNA g-block from IDT (sequence provided in Fig. S1). High-fidelity DNA polymerase was used to PCR amplify the PIC3 cassette with flanking arms that were homologous to the pInducer lentiviral vector (Addgene plasmid #44012). Additionally, sequence for H2B-GFP was similarly PCR amplified from the plasmid hsp70l-loxP-mCherry-STOP-loxP-H2B-GFP_cryaa-cerulean (Hesselson et al., 2009) (Addgene plasmid #24334) using primers with flanking homology arms. These amplicons for PIC3 and for H2B-GFP were ligated into the multiple cloning site (MCS) of the pInducer lentiviral vector using Gibson assembly master mix (NEB E2611) with the H2B-GFP lying downstream of the PIC3 cassette. The *ubiquitin* promoter sequence (Mosimann et al., 2011) and the sequence for the loxP-mCherry-stop-loxP cassette (Addgene plasmid #24334) were similarly PCR amplified and cloned into the pInducer lentiviral vector construct between the MCS and the rtTA site. The sequence for the final construct, *(tre:PIC3-H2B-GFP; ubi:loxP-mCherry-STOP-loxP-rtta)*, was confirmed by sequencing of the entire plasmid.

The assembled construct, *tre:PIC3-H2B-GFP*-*ubi:loxP-mCherry-STOP-loxP*, was co-injected with Tol2 transposase mRNA (Kawakami et al., 2016; Kwan et al., 2007) into zebrafish zygotes to generate multiple independent founders, which were first screened for ubiquitous mCherry expression and then confirmed for H2B-GFP expression upon Cre-mediated recombination. Founders with successful expression of both mCherry and H2B-GFP were propagated further. Transgenic zebrafish larvae were genotyped by epifluorescence at 96 hpf using a Leica M205FA dissecting microscope. Five independent founders were identified, with one founder (F0) having better Cre recombination and the most intense RFP expression. This founder was further propagated and all experiments were performed on the progeny of this founder. In addition, the *Tg(ins:cre)^s924^* established transgenic line was used (Hesselson et al., 2009; Ye et al., 2016). In addition to Cre, this line contains an expression cassette comprised of an *α-crystallin* promoter driving a Venus fluorescent protein; this enables visual genotyping of the line at 48 hpf via fluorescence in the lens. All embryos were collected at spawning and maintained in a 28.5°C incubator in egg water-filled Petri dishes.

### Chemical treatments

1-Phenyl-2-thiourea (PTU; Acros #207250250) supplementation at 0.003% was used to prevent pigmentation in all embryos after gastrulation stages. To determine ideal and reproducible conditions, doxycycline (Sigma-Aldrich #24390-14-5) solutions of 0.5, 1, 2.5, 5, 10 and 20 µg/ml were prepared in egg water (0.1% instant ocean salt, 0.0075% calcium sulfate) that was supplemented with PTU. Some transgene induction was observed at all concentrations ranging from 0.5 µg/ml to 20 µg/ml. Embryos treated with 5 µg/ml doxycycline displayed induction of the pro-inflammatory cytokines without causing islet disaggregation. All embryos from F0 were treated with 5 µg/ml doxycycline for these experiments.

### Immunofluorescence staining and image collection

At the conclusion of each treatment, larvae were washed in egg water and then fixed with 3% formaldehyde in a PEM buffer (0.21 M PIPES, 1mM MgSO_4_, 2 mM EGTA, pH 7) at 4°C overnight. Fixed larvae were washed with PBS and de-yolked, then antibody staining was performed as described (Ye et al., 2015). The following primary antibodies were used: mouse anti-Tnfa (1:50; Abcam #52B83), guinea pig anti-Insulin (1:200; Invitrogen #180067), rabbit anti-Glucagon (1:50; Sigma-Aldrich #SAB4200685) and rabbit anti-Cleaved caspase 3 (1:100; Cell Signaling Technologies #9661S). Primary antibodies were detected with 1:500 dilutions of complementary Alexa Fluor-conjugated secondary antibodies (Jackson ImmunoResearch). After staining, larvae were mounted on slides in VECTASHIELD (Vector Labs H-1000), and confocal imaging was performed with a Zeiss LSM700 microscope. The confocal stacks of pancreatic islets were analyzed with Fiji software (National Institutes of Health). For the macrophage infiltration analysis, GFP^+^ cell quantification was carried out within single-plane optical sections. Macrophages were manually classified as ‘infiltrating’ if they were within the islet and ‘surveilling’ if they were observed within a 100-µm radius extending from the center of the islet.

### qRT-PCR

RNA isolation and reverse transcription was performed using miRNeasy and miScript II RT kits according to the manufacturer's instructions (Qiagen). qRT-PCR was performed using the miScript SYBR Green PCR Kit (Qiagen) and a Mastercycler ep realplex instrument (Eppendorf). Relative RNA levels were established relative to β-actin, using the comparative Ct method (Chakrabarti et al., 2002). RNA primer sequences are listed in Table S1.

### Measurement of free glucose

Glucose colorimetric assays (Bio Vision #K686) and glucose oxidase colorimetric assays (Bio Vision #K86) were performed by pulverizing 20 embryos in each group within 500 µl of assay buffer as described in Andersson et al. (2012) and by following the manufacturer's instructions. Results were measured using a SpectraMax M5 multiwell plate reader (Molecular Devices). Glucose oxidase catalyzes the oxidation of glucose to hydrogen peroxide and D-glucono-δ-lactone, thus its activity is an indicator of free glucose (Ferri et al., 2011). Glucose oxidase activity was determined following the manufacturer's instructions.

### Statistical analysis

Statistical analyses were performed using Prism Version 7.1 (GraphPad Software). The data are presented as the means±s.e.m. Student's *t*-tests were used for comparison between the experimental and control groups. One-way ANOVA with Tukey's post-test for multiple comparisons was used when comparing more than two groups. For all analyses, a *P*-value <0.05 was considered significant. To estimate changes in regional macrophage surveillance over time in multiple embryos, we computed the area under the curve using GraphPad Prism. This measurement was used to compare macrophage activity in CETI-PIC3-induced versus uninduced embryos.

## Supplementary Material

Supplementary information
