# Supplementary material for: A novel Cre-enabled tetracycline-inducible transgenic system for tissue-specific cytokine expression in the zebrafish: CETI-PIC3
Source: Dis Model Mech. 2020 Jun 26;13(6):dmm042556. doi: 10.1242/dmm.042556 (PMC7328138; doi:10.1242/dmm.042556)
Supplement: Supplementary information [file dmm-13-042556-s1.pdf]

Supplementary Materials

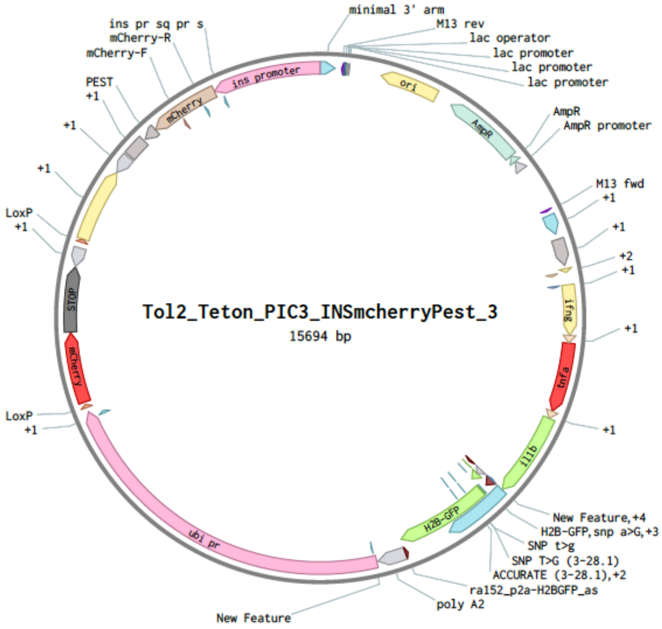

atggattcctgcctcaaaatggtgctactctgtggacttttgtggatagcatctcttcagacaaccagcgcatacagatttcgacggtccaggagcgagaac
ccgattctgaacacaaatatcgagaaactaaaaacacattataacacgcttgcaaaggattgggttggaaaatctgtgtttgtttctcatctggaccagctga
attctaagccaacctgcacttgccaggctgtgttgcttgaaggaatgctgagcatctatgaagacattttccaagacatgatgaacaagtcagataataaag
aagtgagagacgatcttaaaaaagtaattcatgaggtgaaaaatttgaagcacaagtacaacgaagaacataaattatggagggagctccaggatattca
ctcagtcaaggcgaaaaatggaacaatccaagaaagagcattaaatgacttcctgaaggtgtattatcgggcctccacagaaaacggcatttacacatg
GGAGGAGGACAGTGTACTAATTATGCTCTCTTGAAATTGGCTGGAGATGTTGAGAGCAACCC
AGGTCCCGGAGGAGGAatgaagcttgagagtcgggcgcttttggatgttgaagaaggagagttgcctttaccgctggtgatggtgtctagg
aggaaagctggatcttcaaagtcgggtgtatggagggtgtttgggatcattttggctgtgggcctttgtgccgctgctgccgtctgcttcacgctccataaga
cccagggcaatcaacaagatggaagtgtgctgagactcacattgagagatcgcatttcacaaggcaatttcacttccaaggctgccatccatttaacaggt
ggatacaactctgaatcaaagaccttagactggagagatgaccaggaccaggccttttcttcaggtggcttgaaattagtaaacagggagattatcattccc
gatgatggcatttattttgtctacagccaggtgtctttgcacatcagctgcacgtctgaactgactgaggaacaagtgcttatgagccatgcagtgatgcgctt
ttctgaatcctacggaggcaaaaagccacttttcagtgcaatccgctcaatctgcacgcaggagcctgaatctgaaaatctgtggtacaacactatttacctc
ggcgctgccttccatttacgagaaggagacagactgggcacagacacgaccacagcacttctaccgatggttgaaaatgataacggaaagaccttctttg
gggtgtttggtttgGGAGGAGGACAGTGTACTAATTATGCTCTCTTGAAATTGGCTGGAGATGTTGAG
AGCAACCCAGGTCCCGGAGGAGGAatgagaaagcagaggaacttaaccagctctgaaatgatggcatgcgggcaatatgaagt
caccatagctccaaaaaatttgtgggagacagacggtgctgtttattcagattctgatgagatggactgttcagatccgcttgcaatgagctacagatgcga
catgcatgagggcatcaggctggagatgtggacttcgcagcacaaaatgaagcagttggtgaacgtcatcatcgccctgaacagaatgaagcacatcaa
accccaatccacagagtttggagaaaaagaggtgctggacatgctcatggcgaacgtcatccaagagcgtgaagtgaacgtggtggattcagtgccgtc
ttacactaaaaccaaaaacgtcttgcaatgcacgatttgcgatcagtataagaagtctctggtgcgcagcggcggatctccacatttgcaggccgtcacact
gagagccggaagcagcgacttgaaagtgcgcttcagcatgtccacatatgcgtcgcccagtgctccggctacttctgctcagcctgtgtgtttgggaatct
ccaaaagtaacctgtacctggcctgcagccccgccgaaggctccgctccacatctcgtactcaaggagatcagcgggtctctggagaccattaaagctg
gagatccaaacggatacgaccagctgctgttcttcaggaaggagaccggcagctccataaacaccttcgagtccgtcaaatgtcccggttggtttatcag
caccgcgtatgaagattcgcagatggtggagatggacaggaaagacaccgagcgcatcattaacttcgagctgcaggataaagtgcgcatc

Fig. S1. Top: the full plasmid for the CETI-PIC3 line. Bottom: the sequences for the three cytokines with intervening E2A sequences. The sequence for *ifng1* is in yellow. The sequence for *tnfa* is in blue followed by the sequence for *il1b* in purple. The intervening E2A sequences are in green.

Disease Models & Mechanisms • Supplementary information

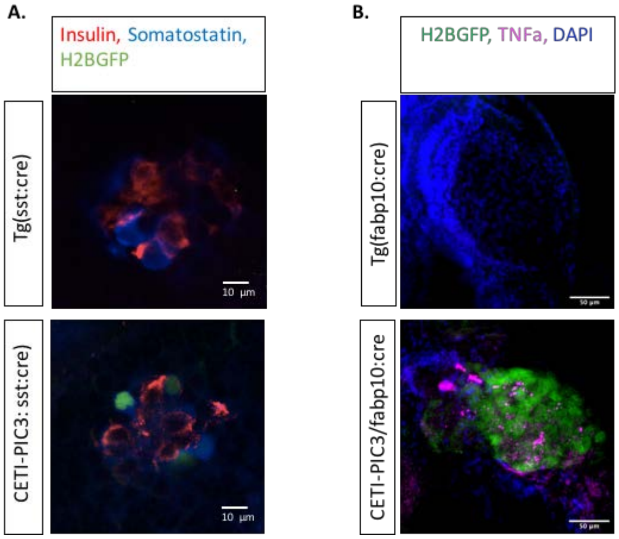

Fig. S2. (A) The CETI-PIC3 line was crossed to a *somatostatin-cre* (*Tg(sst:cre)*) line to induce cytokines in a delta cell-specific manner. (B) The CETI-PIC3 line was crossed to a liver-cre line (*fabp10:cre*) to induce cytokines in a hepatocyte-specific manner.

Disease Models & Mechanisms • Supplementary information

Table S1. The primers used in the qRT-PCR analysis.

Primer Name	Sequence
zf_actin_FWD	CGAGCAGGAGATGGGAACC
Zf_actin_REV	CAACGGAAACGCTCATTGC
Zf_ifng1_FWD	GGGCTTTGCCTGGGGAGTAT
Zf_ifng1_REV	TCAGGATTCGCAGGAAGATGG
ZF_tnfa_FWD	GCGCTTTTCTGAATCCTACG
ZF_tnfa_REV	GTGCCCAGTCTGTCTCCTC
ZF_il1b_FWD	AGGAGACCGGCAGCTCCATA
ZF_il1b_REV	TGATGCGCTCGGTGTCTTTC
ZF_H2BGFP_FWD	GGTACTAAGGCCGTCACCAA
ZF_H2BGFP_REV	GAACTTCAGGGTCAGCTTGC
Zf_nrf2a_FWD	TCACTCCCAGAGTTGCAGCAG
ZF_nrf2a_REV	GTTTGAGCCGAGCCGAGATG
Zf_hmox1a_FWD	GTCAGGGAGCTCACACAGTC
Zf_hmox1a_REV	TTTTCAGCTCTGACGTGGCT
Zf_gpx1a_FWD	AGCATGGCAGGAACCATGAA
Zf_gpx1a_REV	GAAGCCATTTCCAGGACGGA
Zf_sod_FWD	ACCGGCACCGTCTATTTCAA
Zf_sod_REV	AGCATGGACGTGGAAACCAT
ZF_nos2a_FWD	GCCTTGTGCACTCATCTGAA
Zf_nos2a_REV	CCTGCAGGACTAGTGGAAGC
Zf_bip_FWD	GGTCGGCGGCTCCACTCGTAT
Zf_bip_REV	AGCCGGTTCTGGTCGTTGGTAATG
Zf_chop_FWD	GGAGCCCGAGTTTTTGGATGTTTT
Zf_chop_REv	AGTGCGCCGCCTCGTTTTCTT

Disease Models & Mechanisms • Supplementary information
